# Pseudotyped Vesicular Stomatitis Virus-Severe Acute Respiratory Syndrome-Coronavirus-2 Spike for the Study of Variants, Vaccines, and Therapeutics Against Coronavirus Disease 2019

**DOI:** 10.3389/fmicb.2021.817200

**Published:** 2022-01-14

**Authors:** Marcela Salazar-García, Samyr Acosta-Contreras, Griselda Rodríguez-Martínez, Armando Cruz-Rangel, Alejandro Flores-Alanis, Genaro Patiño-López, Victor M. Luna-Pineda

**Affiliations:** ^1^Laboratorio de Biología del Desarrollo y Teratogénesis Experimental, Hospital Infantil de México “Federico Gómez”, Mexico City, Mexico; ^2^Laboratorio de Investigación en COVID-19, Hospital Infantil de México “Federico Gómez”, Mexico City, Mexico; ^3^Laboratorio de Bioquímica de Enfermedades Crónicas, Instituto Nacional de Medicina Genómica, Mexico City, Mexico; ^4^Departamento de Microbiología y Parasitología, Facultad de Medicina, Universidad Nacional Autónoma de México, Mexico City, Mexico; ^5^Unidad de Investigación en Inmunología y Proteómica, Hospital Infantil de México “Federico Gómez”, Mexico City, Mexico

**Keywords:** vesicular stomatitis virus, SARS-CoV-2, Biosafety Level 3, pseudotyped viruses, pseudovirus, glycoprotein, ppVSVΔG-SARS-CoV-2 S

## Abstract

World Health Organization (WHO) has prioritized the infectious emerging diseases such as Coronavirus Disease (COVID-19) in terms of research and development of effective tests, vaccines, antivirals, and other treatments. Severe Acute Respiratory Syndrome-Coronavirus-2 (SARS-CoV-2), the etiological causative agent of COVID-19, is a virus belonging to risk group 3 that requires Biosafety Level (BSL)-3 laboratories and the corresponding facilities for handling. An alternative to these BSL-3/-4 laboratories is to use a pseudotyped virus that can be handled in a BSL-2 laboratory for study purposes. Recombinant Vesicular Stomatitis Virus (VSV) can be generated with complementary DNA from complete negative-stranded genomic RNA, with deleted G glycoprotein and, instead, incorporation of other fusion protein, like SARS-CoV-2 Spike (S protein). Accordingly, it is called pseudotyped VSV-SARS-CoV-2 S. In this review, we have described the generation of pseudotyped VSV with a focus on the optimization and application of pseudotyped VSV-SARS-CoV-2 S. The application of this pseudovirus has been addressed by its use in neutralizing antibody assays in order to evaluate a new vaccine, emergent SARS-CoV-2 variants (delta and omicron), and approved vaccine efficacy against variants of concern as well as in viral fusion-focused treatment analysis that can be performed under BSL-2 conditions.

## Introduction

Infectious diseases develop and re-emerge regularly, triggering epidemics and pandemics (Morens et al., 2008). Human Immunodeficiency Virus/Acquired Immunodeficiency Syndrome (HIV/AIDS; 1981), Nipah virus (1999), Severe Acute Respiratory Syndrome (SARS; 2002), Middle East Respiratory Syndrome (MERS; 2012), and Coronavirus Disease (COVID-19; 2019) are examples of newly emerging infectious diseases while re-emerging infectious diseases have reappeared in new locations, such as West Nile in the United States and Russia (Morens and Fauci, 2020). The World Health Organization’s (WHO) Research and Development Blueprint Initiative has prioritized infection diseases such as COVID-19, Crimean-Congo hemorrhagic fever, Ebola virus and Marburg virus diseases, Lassa fever, MERS and SARS, Nipah and henipaviral diseases, Rift Valley fever, and Zika for the development of effective tests, vaccines, antivirals, and other treatments (Kieny et al., 2016). To be sure, the pathogens that cause these diseases are classified as risk group 3 (high individual risk and low risk to the community) or risk group 4 (high individual risk and high risk to the community, without treatment), which necessitates the use of Biosafety Level (BSL)-3 and BSL-4 laboratories and facilities for handling and propagation (Artika and Ma’roef, 2017).

**FIGURE 1:**
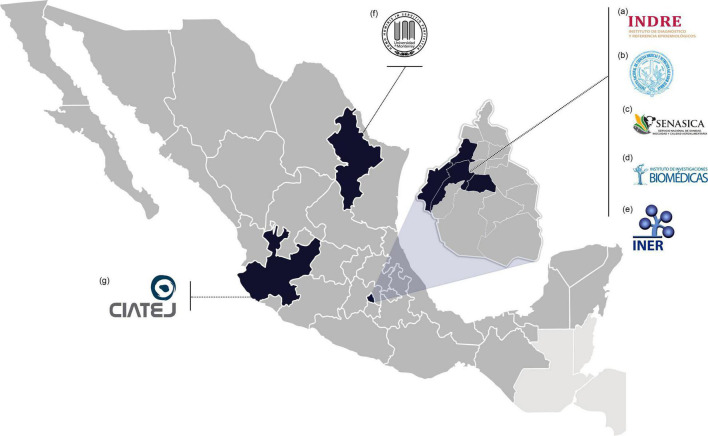
Mexican BSL-3 laboratories. (a) BSL-3 Laboratory from the Institute for Epidemiological Diagnosis and Reference (Mexico City); (b) The Biosecurity Unit from Institute of Biomedical Research (UNAM, Mexico City); (c) Animal Health Laboratory from National Service of Agrifood Health, Safety, and Quality (Mexico City); (d) National Laboratory for Maximum Biological Safety from National Institute of Medical Sciences and Nutrition “Salvador Zubirán” (Mexico City); (e) Emerging and Non-Emerging Pathogens Research Tower from National Institute of Respiratory Diseases (Mexico City); (f) Center for Research and Assistance in Technology and Design of the State of Jalisco (Guadalajara City), and (g) University of Monterrey/Autonomous University of Nuevo León (Monterrey City).

Many nations across the globe lack the infrastructure and resources needed to research these emerging and re-emerging pathogens. There are just seven BSL-3 laboratories in Mexico, and they are typically overworked. From these, the Biosafety Level 3 Laboratory from Institute for Epidemiological Diagnosis and Reference^1^, Biosecurity Unit from Institute of Biomedical Research from UNAM^2^, Animal Health Laboratory from National Service of Agrifood Health, Safety and Quality^3^, National Laboratory for Maximum Biological Safety from National Institute of Medical Sciences and Nutrition “Salvador Zubirán,”^4^ and Emerging and Non-Emerging Pathogens Research Tower from National Institute of Respiratory Diseases^5^ are located in Mexico City, whereas the other two BSL-3: the Center for Research and Assistance in Technology and Design of the State of Jalisco^6^ and the University of Monterrey/Autonomous University of Nuevo León^7^ are located in Guadalajara, Jalisco and Nuevo León, Monterrey, respectively. However, because Mexico has no BSL-4 laboratories, research with WHO-designated priority diseases such as Ebola is hampered (Figure 1).

To enable the study of BSL-3/-4 pathogens under BSL-2 laboratory conditions, one can use pseudotyped virus (PV) to study virus entry (Millet et al., 2019). PV, sometimes known as “pseudoviruses” or “pseudoparticles” (pp), are amplification-defective viruses capable of just one cycle of replication that can infect host cells in the same way as wild-type viruses do (Takada et al., 1997). PVs are primarily derived from retroviruses [HIV and Murine Leukemia Virus (MLV)] and rhabdoviruses (VSV) and are used to investigate the function of viral fusion proteins in enveloped viruses such as lifecycle initiation, host and cellular tropism, interspecies transmission, viral pathogenesis, and host cell entry pathways (Moore et al., 2004; Whitt, 2010; Mendenhall et al., 2012). VSV is a negative polarity enveloped RNA virus with a genome size of 11 kb that contains five main viral proteins: nucleoprotein (N), phosphoprotein (P), matrix protein (M), glycoprotein (G), and large polymerase protein (L). VSV has been frequently utilized as an enveloped virus for the creation of efficient PV harboring a foreign virus’s surface protein.

## Pseudotyped Vesicular Stomatitis Virus: History

Pseudotyped viruses are useful tools for studying the function of viral fusion proteins (Moore et al., 2004; Whitt, 2010; Mendenhall et al., 2012). PVs have been used in phenotypic mixing since the 1970s, in which two encapsulated viruses “share” coat proteins while having distinct genetic material (RNA or DNA) (Choppin and Compans, 1970; Závada, 1972; Huang et al., 1974). Thus, a temperature-sensitive (ts) mutant of VSV was reported, which was deficient in the synthesis of its G protein at nonpermissive temperatures (Schnitzer et al., 1979). Several PVs were supplemented with foreign viral glycoproteins (GPs) using VSV strain ts045, including VSV-Avian sarcoma viruses, VSV-Rous sarcoma virus, VSV-Murine leukemia virus, VSV-Murine oncoviruses and -murine cytomegalovirus, and Rous-associated virus 1 is a VSV-Avian retrovirus (Weiss et al., 1977; Lodish and Weiss, 1979; Schnitzer and Gonczol, 1979; Weiss and Bennett, 1980). Furthermore, an infectious defective interfering (DI) particle was characterized as a VSV strain with faulty replication and amplification of its RNA genome but proper viral packaging (Pattnaik and Wertz, 1990). These studies demonstrated RNAs produced from non-viral origins could be packaged into VSV particles. When co-expressed with the other VSV proteins, a full negative-stranded genomic RNA from a cDNA clone of a VSV DI RNA was replicated, transcribed, and packed into infectious particles (Pattnaik et al., 1992; Stillman et al., 1995). Full-length positive-sense RNA, complementary to the VSV genome, can be produced using the bacteriophage T7 RNA polymerase. Production of the full-length anti-genome along with proteins required for RNA replication (N, P, and L) enable the recovery of replication-competent recombinant (*r*)VSV from DNA plasmids (Lawson et al., 1995; Whelan et al., 1995). Recovery of rVSVs lacking the glycoprotein open reading frame from the genome (ppVSVΔG) is accomplished by supplying the VSV G in *trans* and they are named ppVSVΔG-G (Schnell et al., 1996). Several reporter genes have been cloned into ppVSVΔG permitting various experimental read-outs, and these included green and red fluorescent protein (GFP/RFP/DsRed), secreted Embryonic Alkaline Phosphatase (SEAP), and firefly luciferase (fLuc), generating ppVSVΔG-reporter (Takada et al., 1997; Fukushi et al., 2008; Tani et al., 2010; Muik et al., 2012).

## Importance of the Glycoprotein Cytoplasmic Tail in the Assembly of Pseudotyped Vesicular Stomatitis Viruses

**FIGURE 2:**
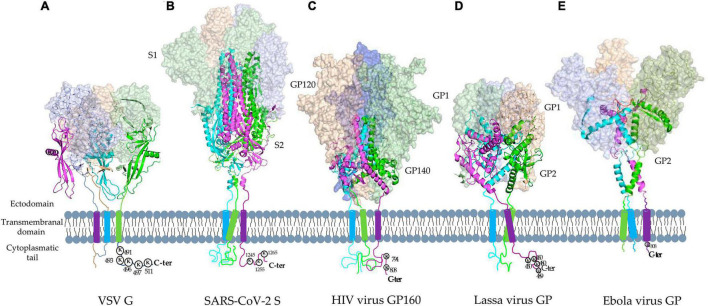
Tridimensional structures of Vesicular Stomatitis Virus G protein and the main class I viral fusion proteins in pre-fusion static conformations. **(A)** Vesicular Stomatitis Virus (VSV) class III fusion glycoprotein (PDB: 6TIT) and representative class I viral fusion proteins: **(B)** SARS-CoV-2 S (PDB: 6VXX), **(C)** HIV glycoprotein (GP) 160 (PDB: 6ULC), **(D)** Lassa virus GP (PDB: 6P91), and **(E)** Ebola virus GP (PDB: 6QD7). They form homotrimers with two domains, the C- and N-terminal domains (Magenta, green, and cyan represent each monomer). N terminal contains the receptor binding site and C terminal domain contains two heptad repeats (HR), a single-pass transmembrane motif, and a cytoplasmic tail (CT).

Enveloped viruses have fusion proteins that enable attachment and fusion into host cells (Barrett and Dutch, 2020). These viral fusion proteins are classified structurally as class I (e.g., HIV Env Glycoprotein), class II (e.g., Rift Valley fever virus glycoprotein C), and class III (e.g., VSV G glycoprotein), with all of them exhibiting both pre- and post-fusion static conformations (Blumenthal et al., 2012; Dessau and Modis, 2013; Kim et al., 2017). Lassa, Ebola, HIV, MERS, SARS, and SARS-CoV-2 viruses all feature class I viral fusion glycoproteins that have two domains, the C- and N-terminal domains located between the furin-like protease cleavage site (Figure 2). In their pre- and post-fusion states, they form homotrimers, and their C terminal domain contains two heptad repeats (HR), a single-pass transmembrane motif, and a cytoplasmic tail (CT) (Muñoz-Barroso et al., 1999; Lee et al., 2008; Hastie et al., 2017; Pallesen et al., 2017; Yuan et al., 2017; Wrapp et al., 2020).

**FIGURE 3:**
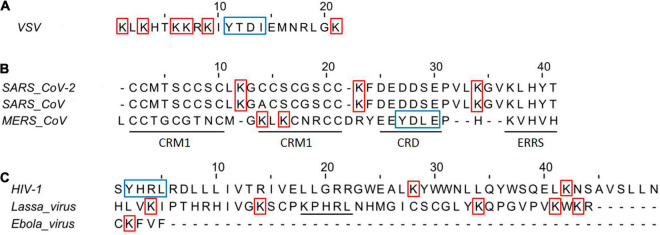
Amino acid sequences of viral glycoproteins class I CT domains. **(A)** Vesicular stomatitis virus CT domain Indian strain. **(B)** Amino acid sequence alignment of CoVs Spike protein in the CT domains. CRM, cysteine-rich motif; CRD, charge-rich domain; ERRS, endoplasmatic reticulum retrieval signal (KxHxx). The amino acid alignment was performed with the Jalview v2.11.1.4 using CLUSTAL W. **(C)** Amino acid of HIV, Lassa, and Ebola virus glycoproteins CT domains. Underline in Lassa virus indicates the ERRS motif. The HIV CT domain was trimmed to 60 and 40 amino acids in the N and C terminal, respectively. The red squares indicate Lys that could be implicated in efficient infectivity, while the blue squares indicate the tyrosine-dependent internalization signals (YxxΦ motif, where Φ is F, I, L, M, or V).

The membrane-associated RING-CH (MARCH)-8, a RING (really interesting new gene)-finger E3 ubiquitin ligase, has been reported to downregulate human transmembrane proteins, including the enveloped viral glycoproteins SARS-CoV-2 spike (S), HIV-1 Env, and EboV-GP (Tada et al., 2015). The CTs found in these viral glycoproteins are made up of Lys residues that can vary in quantity and serve as targets for MARCH-mediated ubiquitination (Figure 3A). Interestingly, expression of MARCH8 in the virus-producing cells reduced the levels of viral glycoprotein and compromised infection of cells was obtained by replacing Lys residues with Ala (K to A) in the CTs of these glycoproteins (Lun et al., 2021). Although S glycoprotein CTs of Coronaviruses (CoVs) features cysteine-rich motifs (six conserved residues) play an important role in S glycoprotein function, these cysteines are palmitoylated and their substitution with Ala (Cys-to-A) affect the S-mediated cell fusion of these viruses (Figure 3; Chang et al., 2000; Petit et al., 2007).

Coronaviruses S CT comprises a dilysine (KKxx-COOH) or a dibasic (KxHxx-COOH) endoplasmic reticulum retrieval signal, as well as a tyrosine-dependent localization signal (YxxF or YxxI motif) that interacts with the CoVsM protein for virions incorporation (Figure 3; Lontok et al., 2004; McBride et al., 2007; Winter et al., 2008; Shirato et al., 2011). CoVs S CT with a nonsense mutation-generated 21- or 24-amino acid deletions (conserved KxHxx motif) showed the highest viral spread and the appearance of non-syncytium-forming infectious centers, likely driving rVSV-SARS-CoV-2 S adaptation for the efficient spread in tissue culture (Case et al., 2020b; Dieterle et al., 2020). In addition, rVSV-Hantaan virus Gn and Gc glycoproteins also acquired a substitution in CT (I532K) of Gn, and a substitution (S1094L) in the stem region of Gc following three serial passages in Vero cells, that have the highest viral spread likely for relocalization of Gn/Gc from the Golgi complex to the cell surface (Slough et al., 2019). Overall, all findings emphasize the relevance of CT of these glycoproteins in the production of virus-like particles and virion, including pseudotyped viruses. Pseudotyped VSV virions have been used to assess cellular tropism, glycoprotein function, receptor recognition, and neutralization antibody assay in viruses from risk groups 3 and 4 (Table 1), including Crimean-Congo hemorrhagic fever virus, Ebola virus, Marburg virus, Lassa fever virus, Nipah virus, Rift Valley virus, MERS-CoV, and SARS-CoV-2 virus (Kunz et al., 2005; Fukushi et al., 2008; Kaku et al., 2012; Bukbuk et al., 2014; Suda et al., 2016; Lennemann et al., 2017; Lester et al., 2019; Saito et al., 2020; Zettl et al., 2020).

**TABLE 1:** Application of pseudotyped Vesicular Stomatitis Virus (VSV).

Virus	Viral protein	Research area and application	Reporter	References
Crimean-Congo hemorrhagic fever Virus	GP	Vaccine, viral entry mechanism, and neutralizing assay	Luciferase	Suda et al., 2016; Rodriguez et al., 2019
Ebola virus	GP	Vaccine and drug testing	GFP	Takada et al., 1997; Geisbert and Feldmann, 2011; Lennemann et al., 2017; Saito et al., 2020
Marburg virus	GP	Vaccine and drug testing	GFP	Geisbert and Feldmann, 2011; Zhang et al., 2017; Saito et al., 2020
Lassa virus	GP	Entry and receptor mechanism, and neutralization assays	GFP	Kunz et al., 2005; Hastie et al., 2017
Nipah virus	G/F	Fusion mechanism and neutralization assays	GFP and SEAP	Kaku et al., 2009, 2012; Tamin et al., 2009; Contreras et al., 2021
Rift Valley virus	GP	Serological assays	Luciferase	Bukbuk et al., 2014
MERS	Spike	Vaccine, neutralization assays, and receptor evaluation	Luciferase and GFP	Fukuma et al., 2015; Liu et al., 2018; Lester et al., 2019
SARS-CoV	Spike	Vaccine, entry mechanism, and neutralization assays	GFP	Fukushi et al., 2006, 2008; Ge et al., 2006; Kapadia et al., 2008
SARS-CoV-2	Spike	Neutralization assays, entry mechanism, treatment testing, vaccine, and vaccine efficacy	GFP, luciferase, and SEAP	Condor Capcha et al., 2020; Gasbarri et al., 2020; Collier et al., 2021; Malherbe et al., 2021; Tolah et al., 2021; Verma et al., 2021

*GP, glycoprotein; GFP, green fluorescent protein; SEAP, secreted alkaline phosphatase; F, fusion; G, attachment.*

## Pseudotyped Vesicular Stomatitis Virus-Severe Acute Respiratory Syndrome-Coronavirus-2 Spike Protein

Pneumonia caused by the SARS-CoV-2 virus was recognized as COVID-19 by the WHO and is now considered a pandemic, with over 200 million cases and over four million fatalities globally^8^. More than three million COVID-19 infections have been reported in Mexico, with over 250 thousand deaths^9^. Because of the SARS-CoV-2 virus’s high infectivity, toxicity, and lack of therapies, BSL-3 laboratories are necessary for its handling in drug testing, neutralizing antibodies, and authorized vaccination effectiveness (Kaufer et al., 2020). As a result, most research laboratories have been unable to conduct SARS-CoV-2 research; however, the use of ppVSVΔG-SARS-CoV-2 S provides a feasible option for studying this virus at BSL-2 facilities (Plescia et al., 2021).

**FIGURE 4:**
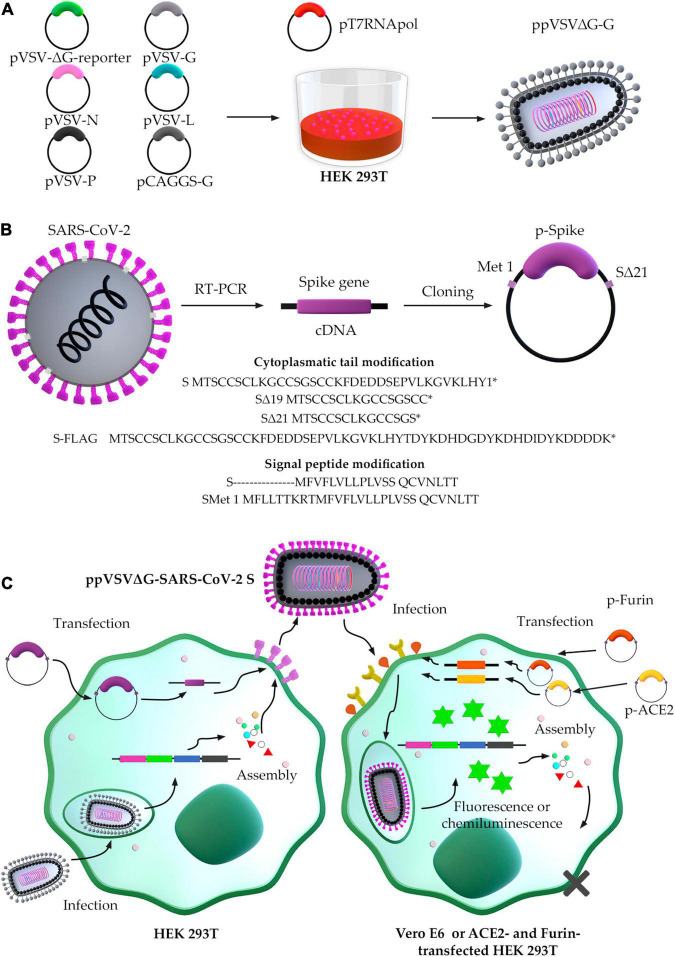
Scheme of the generation of pseudotyped vesicular stomatitis virus-severe acute respiratory syndrome-coronavirus-2 (SARS-CoV-2) S. **(A)** Packaging of ppVSV. **(B)** Optimized conditions for cloning Spike gene. **(C)** ppVSVΔG-SARS-CoV-2 S assembly in HEK 293T cells, and infection assay in Vero E6 cells by ppVSVΔG-SARS-CoV-2 S.

The generation of ppVSVΔG-SARS-CoV-2 S may be classified as (a) ppVSVΔG packaging and (b) SARS-CoV-2 S protein production (Figure 4). The ppVSVΔG is a viral particle that can be packaged *in vitro* by transfection of six plasmids into the bacteriophage T7 RNA polymerase-harboring cell. Although these particles lack a glycoprotein in their genome, they are coated in VSV-G in *trans* that result in ppVSVΔG-G (G-complemented particles). In a new passage of cells, it may incorporate the SARS-CoV-2 S protein onto the virus’s surface by cotransfection with the S gene-containing plasmid, producing ppVSVΔG-SARS-CoV-2-S particles or pseudotyped VSV-SARS-CoV-2-S (Lawson et al., 1995; Whelan et al., 1995; Schnell et al., 1996).

### Vesicular Stomatitis Virus-ΔG Pseudoparticles Packing

In primary transfection, five plasmids are used to accomplish ppVSVΔG packaging: (1) pVSV-ΔG-reporter containing all VSV antigenome directed by the T7 promoter, excepting the GP gene, which was deleted and replaced by a reporter gene-GFP, -RFP/DsRed, -SEAP, or -fLuc; (2) pVSV-G containing the VSV GP gene directed by the T7 promoter; (3) pVSV-L containing the VSV polymerase (L) gene directed by T7 promoter; (4) pVSV-N containing VSV nucleocapsid (N) gene directed by T7 promoter; and (5) pVSV-P containing VSV phosphoprotein (F) gene directed by T7 promoter. During cellular passages, ppVSVΔG-G is generated with pCAGGS-G containing VSV GP gene directed by pol II promoter as chicken beta-actin promoter (Figure 4A).

To be able to transcribe the VSV antigenome and the N, P, G, and L genes, the HEK293T cell line must first be infected with the vaccinia virus or transfected with pT7pol (expressing the bacteriophage T7 RNA polymerase). The VSV polymerase complex (VSV L and P proteins) and the envelope nucleoprotein N produce genome from the antigenome RNA produced by T7. The matrix (M) protein is encoded in the VSV RNA genome and is produced after the genome is made and transcription occurs. Because the genome lacks glycoprotein, the cell line must be transfected with pCAGGS-G to produce G in *trans*, and make infectious G pseudotyped ppVSVΔG-G virions (Figure 4A; Whitt, 2010).

### Production of Severe Acute Respiratory Syndrome-Coronavirus-2 S Protein: Optimized S Gene-Containing Plasmid

The SARS-CoV-2 S protein interacts with the host cell receptor, allowing viral and cellular membranes to fuse. The SARS-CoV-2 S protein comprises a signal peptide, an N-terminal S1 domain, and a C terminal S2 domain and has 1,274 amino acids. Replacing rare codons with abundant cognate tRNA in the cytosol can affect protein translation from mRNA, resulting in guanine and cytosine enrichment as a process of sequence optimization with higher steady-state mRNA levels *in vitro* and protein expression *in vivo* (Kudla et al., 2006; Mauro and Chappell, 2014). Furthermore, the SARS-CoV-2 S protein comprises a short signal peptide with weak Sec61 recognition, but the inclusion of the nine upstream residues improves recognition and increases protein levels (Havranek et al., 2020).

Severe Acute Respiratory Syndrome-Coronavirus-2 isolates from the initial human cases in Wuhan had an S protein with the D614 form; however, the viruses that are currently circulating in the human population have the G614 form. SARS-CoV-2 viruses with the G614 mutation in the S protein have greater infectious titers *in vitro* than those with the D614 mutation (Korber et al., 2020). Another R682Q mutation in the S protein has been characterized as a fast adaptation of SARS-CoV-2 after successive passaging in Vero E6 cells (Ogando et al., 2020). Both the D614G and R682Q alterations improve VSV-SARS-CoV-2 pseudotyping, with the S protein containing 19 deleted residues into the C terminus (19) (Johnson et al., 2020). CT alterations of various viral envelope GPs, including truncation and point mutation in this tail, may be necessary to facilitate appropriate integration into the ppVSVΔG-SARS-CoV-2 S. The greater incorporation into ppVSVΔG-SARS-CoV-2 S is most likely related to a change in cellular localization that produces additional S at sites of assembly (Zingler and Littman, 1993; Mammano et al., 1997; Fukushi et al., 2006; Slough et al., 2019). When compared to WT Spike, first research utilizing shortened SARS-CoV-2 S protein with a deletion (Δ) of 8 to 39 amino acids in CT demonstrated a high titer of ppVSVΔG-SARS-CoV-2 S in Δ19 and Δ26 (Giroglou et al., 2004). When the deletions Δ19 and Δ21 into CT were examined, additional investigations revealed a high titer of ppVSVΔG-SARS-CoV-2 S with increased cell-to-cell fusion (Case et al., 2020b; Dieterle et al., 2020; Havranek et al., 2020; Johnson et al., 2020; Schmidt et al., 2020). If necessary, the SARS-CoV-2 S protein can be fused with a C terminal 3XFLAG tag to detect full-length S without interfering with fusion, surface expression, translation, and VSV incorporation of SARS-CoV-2 S protein (Havranek et al., 2020). All findings have proposed the cloning of Met1-S-Δ21 with usage codons for efficient production of SARS-CoV-2 S protein and ppVSV assembly into the optimal cell line (Figure 4B). The obtained ppVSV viral particles are used to infect a cell line previously transfected with the plasmid containing the optimized S gene that will provide S protein for its assembly onto the virus’s surface.

### Optimal Cell Line to Evaluate of Pseudotyped Vesicular Stomatitis Virus-Severe Acute Respiratory Syndrome-Coronavirus-2 Spike Entry

By attaching to a cellular receptor, like the angiotensin-converting enzyme 2 (ACE2), the SARS-CoV-2 S protein promotes viral entry into target cells (Datta et al., 2020; Wrapp et al., 2020). The lungs and other tissues, including the nasal and oral mucosa, vasculature, kidney, heart, gastrointestinal tract, pancreas, and brain, express ACE2 (Gembardt et al., 2005). Thus, cell lines from human and animal origin, such as 293T (human kidney cells), BHK-21 (Syrian hamster kidney cells), Huh-7 (human liver cells), LLC-PK1 (pig kidney cells), MRC-5 (human lung cells), MyDauLu/47.1 [Daubenton’s bat (Myotis daubentonii) lung cells], NIH/3T3 (Mouse embryonic fibroblast cells), RhiLu/1.1 [Halcyon horseshoe bat (Rhinolophus alcyone) lung cells], Vero (African green monkey kidney cells), Calu-3 (human lung cells), Caco-2 (human colon cells), MDBK (cattle kidney cells), MDCKII (Dog kidney cells), A549 (human lung cells), BEAS-2B (human bronchus cells), and NCI-H1299 (human lung cells) have been evaluated for ppVSVΔG-SARS-CoV-2 S entry (Hoffmann et al., 2020). Preliminary investigations revealed that Vero cells were highly susceptible to ppVSVΔG-SARS-CoV-2 S, along with Caco-2 and Calus-3, while other cell lines tested were not efficient for ppVSVΔG-SARS-CoV-2 S entry (Hoffmann et al., 2020). Vero E6 cells were also initially utilized in cell-culture-based infection models for SARS-CoV-1 studies (Keyaerts et al., 2005; Yamate et al., 2005). Several studies have compared different cell lines for ppVSVΔG-SARS-CoV-2-S entry efficiency. When ppVSV-SARS-CoV-2-S was titrated in both Vero E6 and MA104 (Monkey African Green kidney) cell lines, the virus entry was increased (Case et al., 2020b). In addition, transfecting non-susceptible 293T cells with ACE2, Furin, and TMPRSS2 greatly enhanced the entry of ppVSV-SARS-CoV-2-S particles, supporting the fact that ACE2, Furin, and TMPRSS2 are required for optimal spike infectivity of kidney cells (Condor Capcha et al., 2020; Johnson et al., 2020; Xiong et al., 2020).

## Pseudovirus Neutralization Assay: Safety, Speed, and Scalable

Although the enzyme-linked immunosorbent test is extensively used for detecting SARS-CoV-2 specific antibodies, it does not offer information on virus-neutralizing antibody titers (Roy et al., 2020). The neutralization test is a technique for determining the presence of neutralizing antibodies. The traditional viral neutralization experiment necessitates the use of a live SARS-CoV-2 virus, which must be handled in a BSL-3 facility. The downside of using a live viral test is that it is labor-intensive, taking up to 4 days to complete (Ogando et al., 2020). To address these challenges, the pseudovirus neutralization assay (PVNA) is a viable option since it can be conducted under BSL-2 settings and is a safe, rapid, and scalable test. ppVSVΔG-SARS-CoV-2 S can be kept for an extended period (≥6 months) with negligible titer loss at −20 or −80, 4°C (4 weeks), or even at room temperature (1 week). Furthermore, ppVSV-SARS-CoV-1-S may withstand many freeze-thaw cycles without decreasing their infectivity (Wright et al., 2009; Molesti et al., 2014). Another advantage of using PVNA is that most ppVSVΔG-SARS-CoV-2 S contain a marker gene that can be detected by a fluorescence or luminescence signal, with a linear correlation between the value of fluorescence or chemiluminescence and the number of infected ppVSVΔG-SARS-CoV-2 S, allowing for easier and more accurate quantification (Case et al., 2020b; Nie et al., 2020a).

### Material and Equipment

**FIGURE 5:**
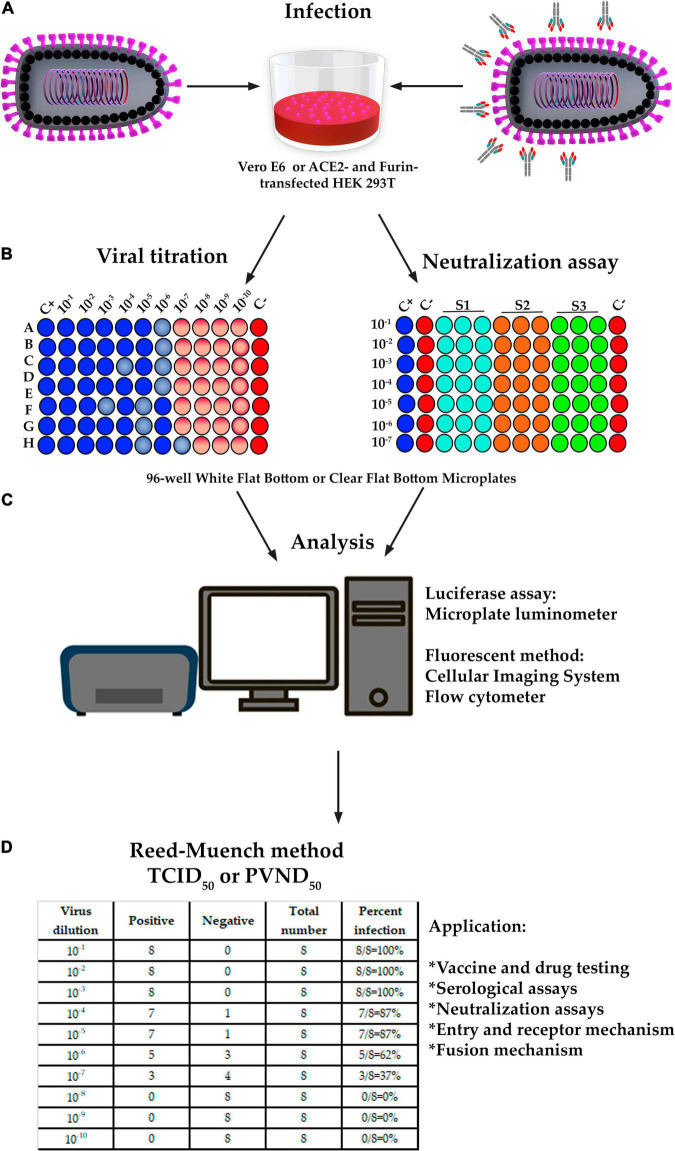
The design of titration and pseudovirus neutralization assay (PVNA). **(A,B)** Infection, titration and PVNA was performed in a 96-well plate with 10-fold serial dilution of ppVSVΔG-SARS-CoV-2 S until a total of 8 or 10 dilutions and 6 or 8 replicates were obtained. Neutralization assay was also performed in a 96-well plate with 6 dilutions and 2 replicates; however, the sera samples were previously diluted and then mixed with ppVSVΔG-SARS-CoV-2 S 325 to 1,300 TCID_50_/mL and incubated for 1 h at 37°C. **(C)** In titration, the cells containing the luciferase were lysed to perform the luciferase assay and a 96-well-plate luminometer was used. In the fluorescence method, the DAPI-stained cells containing GFP were analyzed by fluorescence microscope, and the images were analyzed with specialized software. An alternative method is by using a flow cytometer equipped with a 96-well-autosampler. In PVNA, the luciferase activity was determined by the relative light units (RLU) and the fluorescence by the number of GFP-positive cells. Percent neutralization must be normalized considering uninfected cells as 100% neutralization and infected cells with ppVSVΔG-SARS-CoV-2 S alone as 0% neutralization. **(D)** The 50% tissue culture infectious dose (TCID_50_) and/or the 50% ppVSVΔG-SARS-CoV-2 S neutralizing doses (PVND_50_) were calculated according to the Reed-Muench method and reference table.

Microplate luminometer is a sensitive, ready-to-use device for gene reporter, cell-based, and biochemical experiments in 96-well plates with luminous responses (Alam and Cook, 1990). Fluorescent imaging and quantitation may be assessed using a variety of microscopes that must automatically scan 96-well plates and include quick focusing, image collection, and big data processing (Case et al., 2020b). Flow cytometers can also be used to assess fluorescent-based techniques. ppVSVΔG-SARS-CoV-2 S titration is performed in 96-well plates with 10-fold serial dilution until a total of nine dilutions with six duplicates are obtained (Figure 5; Crawford et al., 2020).

### Calculation of Tissue Culture Infectious Dose or Pseudotyped Virus Neutralization Doses

The Reed-Muench method is used to determine the 50% tissue culture infectious dose (TCID_50_) or pseudotyped virus neutralization doses (PVND_50_) of the ppVSVΔG-SARS-CoV-2 S, which includes calculating the proportional distance (PD) between dilutions above and below the 50% endpoint (Reed, 2010; Gauger and Vincent, 2014; Manenti et al., 2020). The dilution factor is defined as the fold difference between two inoculum titers that are above and below a 50% response rate. Based on the results of Figure 5, the equation must be applied as follows:


(1) PD=(50%)-(%ofinfectedatdilutionnextbelowat  50%)(%ofinfectednextaboveat  50%)-(%ofinfectednextbelowat  50%)

(2) logID50=(logofdilutionnextbelowat  50%)+(PD×logofdilutionfactor)

(3) $${\text{ID}}_{50}=10^{{\text{ID}}_{50}}$$

(4) TCID50mL=inverseofID50/inoculumvolumeinmL×numericalfactortoreach  1mL

### Neutralization Assay and Correlation

Sera samples must be diluted before being combined with ppVSVΔG-SARS-CoV-2 S 325 to 1,300 TCID50/mL and incubated at 37°C for 1 h. Pre-incubated ppVSVΔG-SARS-CoV-2 S are used to infect cell lines for 24–72 h at 37°C and 5% CO_2_, and the quantification of ppVSVΔG-SARS-CoV-2 S infecting the target cells is estimated by measuring the production of luciferase or fluorescence as stated in the section “Pseudotyped Vesicular Stomatitis Virus-Severe Acute Respiratory Syndrome-Coronavirus-2 Spike titration” (Figure 5B). The luciferase activity is determined by relative light units (RLU), whereas the fluorescence is determined by the number of GFP-positive cells. Percent neutralization must be adjusted by assuming uninfected cells to be 100% neutralized and infected cells with ppVSVΔG-SARS-CoV-2 S alone to be 0% neutralized (Crawford et al., 2020). The PVND50 (50% ppVSVΔG-SARS-CoV-2 S neutralizing doses) is determined as described in the section “Pseudotyped Vesicular Stomatitis Virus-Severe Acute Respiratory Syndrome-Coronavirus-2 Spike Titration” (Reed, 2010; Gauger and Vincent, 2014; Manenti et al., 2020).

For some groups, there is a good connection between the experimental data acquired using authentic SARS-CoV-2 and the PVNA. Neutralizing activity was detected with a remarkable connection between the two tests, as demonstrated by Spearman’s correlation with *R* values ranging from 0.76 to 0.939 and *p* values of <0.001 (Case et al., 2020b; Dieterle et al., 2020; Hyseni et al., 2020; Nie et al., 2020a; Xiong et al., 2020; Zettl et al., 2020; Tolah et al., 2021). Furthermore, once a ppVSVΔG-SARS-CoV-2 S stock is produced, the PVNA may be completed in 1 day with a GFP or RLU readout of 7.5 h, whereas the live SARS-CoV-2 assay takes 30 h (Case et al., 2020b; Nie et al., 2020b).

## Pseudotyped Vesicular Stomatitis Virus-Severe Acute Respiratory Syndrome-Coronavirus-2 Spike and Its Use

### New Variants of Severe Acute Respiratory Syndrome-Coronavirus-2

A SARS-CoV-2 variant is identified by alterations in receptor binding, decreased antibody neutralization (post-infection or post-vaccination), decreased treatment effectiveness, or even a possible influence on diagnostics, as well as increased anticipated transmissibility or disease severity (WHO). ppVSVΔG-SARS-CoV-2 S particles can rapidly be produced with the changes in newly define variants and tested for alterations in neutralizing capacity and entry efficiencies.

South Africa was the first to describe the SARS-CoV-2 B.1.351 variety (Beta), which was characterized by N501Y, K417N, and E484K alterations. This variant is more transmissible and resistant to neutralizing antibodies. ppVSVΔG-SARS-CoV-2 S containing the S protein (B.1.351)-associated mutations can quickly infect cell cultures and tolerate neutralizing action of monoclonal antibodies against SARS-CoV-2 S RBD (Kim et al., 2021).

The SARS-CoV-2 B.1.427/B.1.429 variant (epsilon), a novel variant with S13I, W152C, and L452R amino acid changes, was reported for the first time in California (34 nations, beginning May 2021) with enhanced transmissibility and infectivity. When compared to the D614G change, ppVSVΔG-SARS-CoV-2 S bearing the L452R change showed a 6.7- to 22.5-fold higher infectivity in cell cultures and lung organoids, while the W152C change showed just a little increase in infection in these cells. Furthermore, ppVSVΔG-SARS-CoV-2 S with S13I, W152C, and L452R changes demonstrated considerable resistance to neutralization by post-infection (4.0- to 6.7-fold) and vaccination-elicited antibodies (2-fold) as well as monoclonal antibodies (Deng et al., 2021; McCallum et al., 2021).

In the New York region, the SARS-CoV-2 B.1.526 variation (Iota), defined by L5F, T95I, D253G, E484K, or S477N, D614G, and A701V change, was identified. ppVSVΔG-SARS-CoV-2 S harboring L5F, T95I, D253G, E484K, D614G, and A701V, as well as L5F, T95I, D253G, S477N, D614G, and Q957R changes, showed reduced neutralization by post-infection- (2- to 4.5-fold) and vaccine-elicited antibodies (2- to 6-fold) (West et al., 2021).

Recently, a new SARS-CoV-2 VOC was identified in November 2021 and named as Omicron (B.1.1.529) variant by the WHO^10^. This variant was first detected in Botswana (Gauteng Province) on November 11, 2021, and 3 days later in South Africa. The Omicron variant is characterized by 26–32 changes in the S protein, particularly within the RBD, as well as three deletions and one insertion in the S protein, along with mutations outside of the S protein [Network for Genomic Surveillance in South Africa (NGS-SA)]. Many of these mutations are either known or predicted to contribute not only to increase infectivity and transmissibility, but also to confer therapeutic and neutralization resistance (Schmidt et al., 2021). Indeed, Pulliam et al. (2021), reported that reinfections in South Africa have increased as Omicron has spread. Therefore, studies comparing the SARS-CoV-2 Omicron variant vs. ppVSVΔG-SARS-CoV-2 S are urgently needed to get a better understanding of how the immune system is reacting to this variant.

We propose comparing the SARS-CoV-2 B.1.617.2 (delta) variant to ppVSVΔG-SARS-CoV-2 S with L452R, D614G, and P681R amino acid changes. This variety, which is the most common SARS-CoV-2 strain in Mexico and across the world, necessitates immediate monitoring for vaccination effectiveness. In addition, given the fast spread of the Omicron variant, we also propose to compare the SARS-CoV-2 Omicron variant to ppVSVΔG-SARS-CoV-2 S.

### Coronavirus Disease Vaccine

The ongoing development of SARS-CoV-2 variants of concern (VOC) across the world emphasizes the need of monitoring the effectiveness of approved vaccinations for human use. So, far, six very effective COVID-19 vaccines have been approved for human use: Moderna mRNA1273, BioNTech BNT162b2, Janssen Ad26.COV2.S, Gamaleya’s Sputnik V, AstraZeneca’s AZD1222, and CoronaVac. All of these vaccines, albeit in various forms, are based on the Spike protein. Because VOC contains polymorphisms on the S gene, there is an urgent need to evaluate vaccination effectiveness against prevalent SARS-CoV-2 VOC in all geographic areas. As a result, ppVSVΔG-SARS-CoV-2 S have played an important role in determining vaccination effectiveness for SARS-CoV-2 VOC. ppVSVΔG-SARS-CoV-2 S, in particular, has been frequently used in assessing neutralizing antibody titers of vaccinated persons to evaluate if vaccinations provide enough protection against SARS-CoV-2 VOC infection (Table 2; Collier et al., 2021; Ikegame et al., 2021; McCallum et al., 2021; Muik et al., 2021; Shen et al., 2021a,b; Wang G.-L. et al., 2021; Wang P. et al., 2021).

**TABLE 2:** Summary of post-vaccine sera evaluated for neutralization potency by using pseudotyped VSV-Severe Acute Respiratory Syndrome-Coronavirus-2 (SARS-CoV-2) Spike variants of concern (VOC).

Vaccine	Company	Spike construct	Number of samples	Time of sample collection	B.1.1.7^a^ (Alpha)	P.1^a^ (Gamma)	B.1.351^a^ (Beta)	B.1.429^a^ (Epsilon)	References
BNT162b2 BNT162b2	Pfizer/BioNTech Pfizer/BioNTech	2P 2P	37 21	3 weeks after 1st boost 3 weeks after 2nd boost	3.2-fold decrease 1.9-fold decrease	ND ND	ND ND	ND ND	Collier et al., 2021
mRNA-1273 NVX-CoV2373	Moderna Novavax	2P 3Q-2P	29 28	28 days after 2nd boost 2 weeks after 2nd boost	1-3-fold decrease 1-3 fold decrease	ND ND	ND ND	ND ND	Shen et al., 2021b
BBIBP-CorV CoronaVac	Sinopharm Sinovac	Native Native	25 25	2-3 weeks after 2nd boost 2-3 weeks after 2nd boost	Unchanged 0.7-fold change	ND ND	2.5-fold change 3.3-fold change	ND ND	Wang G.-L. et al., 2021
mRNA-1273 NVX-CoV2372	Moderna Novavax	2P 3Q-2P	26 23	28 days after 2nd boost 14 days after 2nd boost	ND ND	ND ND	9.7-fold decrease 14.5-fold decrease	2- fold decrease 2.5-fold decrease	Shen et al., 2021a
Sputnik V	Gamaleya	Native	12	1 month after 2nd boost	Unchanged	2.1-fold decrease	6.1-fold decrease	ND	Ikegame et al., 2021
mRNA-1273 BNT162b2	Moderna Pfizer/BioNTech	2P 2P	12 10	15 days after 2nd boost 7 days after 2nd boost	Unchanged Unchanged	ND ND	12.4-fold decrease 10.3-fold decrease	ND ND	Wang P. et al., 2021
BNT162b2 BNT162b2	Pfizer/BioNTech Pfizer/BioNTech	2P 2P	26 (23-55 year-old) 14 (57-73-year-old)	29 days after 2nd boost 43 days after 2nd boost	0.78-fold decrease 0.83-fold decrease	ND ND	ND ND	ND ND	Muik et al., 2021
mRNA-1273 BNT162b2	Moderna Pfizer/BioNTech	2P 2P	15 18	7-27 days after 2nd boost 7-27 days after 2nd boost	ND 1.3-fold reduction	ND 1.7-fold decrease	ND 3.2-fold decrease	2.2-fold decrease 2.9-fold decrease	McCallum et al., 2021

*^a^Neutralization assay (IC50 fold reduction compared to WT) in VOC, ND, not determined.*

Overall, the data show that SARS-CoV-2 VOC can reduce neutralization potency in vaccinated people’s sera. To note, the SARS-CoV-2 beta variant exhibited the greatest decrease in PVNAs in sera from persons who had been vaccinated with Moderna, Novavax, or PfizerBioNTech (Table 2). Despite the apparently worrying results obtained in the neutralization tests, it is important to consider that vaccines also induce cell mediated immunity; thus, even when neutralization levels decrease, vaccine effectiveness is still high. As a result, monitoring the neutralizing activity evoked by vaccine sera will be required to assess whether a vaccination update is required to limit the establishment and spread of new SARS-CoV-2 variations, such as the delta and omicron variants. Furthermore, ppVSVΔG-SARS-CoV-2 S are being utilized in the development of a vaccine against SARS-CoV-2. Thus, various VSV-SARS-CoV-2 vaccines have been shown in animal models to be effective in both generating neutralizing antibodies at high titers and protecting against the SARS-CoV-2 challenge (Case et al., 2020a; Yahalom-Ronen et al., 2020; Lu et al., 2021; Malherbe et al., 2021). Although the evidence provided by these vector vaccines is encouraging, it is still early in the research process, and additional study is needed before moving forward with human trials.

### Coronavirus Disease Therapeutics

Some COVID-19 treatment drugs are directed against the SARS-CoV-2 S protein and its receptor ACE2, which are found on the membranes of different human cells. Through ACE2 attachment, SARS-CoV-2 causes cell-membrane fusion, facilitating viral entrance (Hoffmann et al., 2020). Although preclinical research for effective drugs against SARS-CoV-2 necessitates the use of live SARS-CoV-2, ppVSVΔG-SARS-CoV-2 S can be utilized for S protein-focused therapy evaluation by blocking or down-regulating ACE2 or preventing viral fusion.

MEK inhibitors (VS-6766, trametinib, and selumetinib) have been used to reduce ACE2 cellular expression as a method to prevent early SARS-CoV-2 infection. ppVSVΔG-SARS-CoV-2 S and human bronchial epithelial, small airway epithelial, and lung cancer cells were utilized to assess infectivity alterations caused by MEK inhibitors (Zhou et al., 2020). Furthermore, PVs were used to test the creation of fusion inhibitor peptides against SARS-CoV-2, and micromolar concentrations of peptides suppressed ppVSVΔG-SARS-CoV-2 S infection by inhibiting viral fusion (Kandeel et al., 2021). Demethylzeylasteral, another inhibiting drug, can interact with hACE2 and the RBD of the SARS-CoV-2 S protein, thus when tested in ppVSVΔG-SARS-CoV-2 S, it inhibited ppVSVΔG-SARS-CoV-2 S entrance into 293T cells (Zhu et al., 2020). Polyunsaturated-3 fatty acids limit SARS-CoV-2 binding and cellular entrance, while linolenic and eicosapentaenoic acids prevent ppVSVΔG-SARS-CoV-2 S penetration (Goc et al., 2021). Inhalable nano catchers containing hACE2 are a proposal for SARS-CoV-2 suppression, which was tested with ppVSVΔG-SARS-CoV-2 S and shown a strong capability for infection inhibition in a hACE2-expressing mouse model (Zhang et al., 2021). Furthermore, when tested with ppVSVΔG-SARS-CoV-2 S and live SARS-CoV-2, hACE2-Fc inhibited Vero E6 cells (Case et al., 2020b).

## Conclusion

There are only seven BSL-3 laboratories in Mexico, four of which are in Mexico City. Because of the scarcity of BSL-3 facilities, research into existing and newly emerging infectious diseases are hampered. Furthermore, there is no BSL-4 laboratory in Mexico, making it impossible to research the viruses that are classified as risk group 4 and are designated priority diseases by the WHO. This is an invitation to the Mexican government to develop research policies and infrastructure for the construction of BSL3 and BSL4 facilities. ppVSVΔG-SARS-CoV-2 S is an excellent choice for studies of host and cellular tropism, interspecies transmission, viral pathogenesis, and host cell entry pathways with risk group 3 and 4 viruses such as SARS-CoV-2. It can be used in BSL-2 facilities for studies of host and cellular tropism, interspecies transmission, viral pathogenesis, and host cell entry pathways. ppVSVΔG-SARS-CoV-2 S can be supplemented with SARS-CoV-2 S protein via changes that improve expression efficiency, and we propose the cloning of Met1-S-Δ21 with usage codons. Once the S gen has been refined and cloned, we propose using site-directed mutagenesis to generate novel variations of the SARS-CoV-2 S protein. ppVSVΔG-G and ppVSVΔG-SARS-CoV-2 S particles must be produced in HEK 293T cells, and infection assays with ppVSVΔG-SARS-CoV-2 S in Vero E6 or ACE2- and Furin-transfected HEK 293T cells are suggested. The ppVSV-SARS-CoV-1-S are stable once produced, and they may be kept with low activity loss for up to 6 months (−20 or −80°C), 4 weeks (4°C), and just 1 week (room temperature) with up to four freeze-thaw cycles. PVNA, surprisingly, is a safe (BSL-2), efficient (7.5 h), and scalable (*r* ≥ 0.9 and *p* ≤ 0.001) method that may be utilized to evaluate novel SARS-CoV-2 variants, post-infection- and vaccine-elicited neutralizing antibodies, and S protein-based COVID-19 therapies. ppVSVΔG-SARS-CoV-2 S can be detected by a fluorescence or luminescence signal with a linear correlation between the value of fluorescence or chemiluminescence and the number of infective ppVSVΔG-SARS-CoV-2 S, and these particles can be easily calculated using the TCID50/mL or PVID50 equations, which are included in this review. We are the first Mexican organization to employ ppVSVΔG-SARS-CoV-2 S for preclinical assessment of post-infection-neutralizing antibodies, evaluation of a possible COVID-19 vaccine and decontamination equipment, and evaluation of vaccinated volunteers against SARS-CoV-2 delta variants.

## Author Contributions

VL-P and MS-G: conceptualization, resources, project administration, and funding acquisition. AC-R and AF-A: software and formal analysis. SA-C, GP-L, GR-M, AC-R, and VL-P: investigation. VL-P, GP-L, and GR-M: writing—original draft preparation. VL-P, GP-L, and MS-G: writing—review and editing. SA-C, AC-R, and AF-A: visualization. VL-P: supervision. All authors have read and agreed to the published version of the manuscript.

## Conflict of Interest

The authors declare that the research was conducted in the absence of any commercial or financial relationships that could be construed as a potential conflict of interest.

## Publisher’s Note

All claims expressed in this article are solely those of the authors and do not necessarily represent those of their affiliated organizations, or those of the publisher, the editors and the reviewers. Any product that may be evaluated in this article, or claim that may be made by its manufacturer, is not guaranteed or endorsed by the publisher.

## Footnotes

1
https://www.gob.mx/salud/acciones-y-programas/instituto-de-diagnostico-y-referencia-epidemiologicos-indre

2
https://www.biomedicas.unam.mx/servicios/laboratorio-de-alta-seguridad-bsl-3/

3
https://www.gob.mx/senasica/acciones-y-programas/laboratorio-de-la-comision-mexico-estados-unidos-para-la-prevencion-de-la-fiebre-aftosa-y-otras-enfermedades-exoticas-de-los-animales-cpa

4
https://www.incmnsz.mx/imagenes/siteLaboratoriodeMicrobiologia/laboratoriobsl3.html

5
https://www.gob.mx/salud/iner

6
https://ciatej.mx/

7
https://www.udem.edu.mx/es/ciencias-de-la-salud/noticia/cuenta-con-recertificacion-internacional-laboratorio-de-bioseguridad

8
https://coronavirus.jhu.edu/map.html

9
https://datos.covid-19.conacyt.mx/

10
https://www.who.int/news/item/26-11-2021-classification-of-omicron-(b.1.1.529)-sars-cov-2-variant-of-concern
